# Local connections of excitatory neurons in motor-associated cortical areas of the rat

**DOI:** 10.3389/fncir.2013.00075

**Published:** 2013-05-28

**Authors:** Takeshi Kaneko

**Affiliations:** Department of Morphological Brain Science, Graduate School of Medicine, Kyoto UniversityKyoto, Japan

**Keywords:** local circuit, microcircuit, pyramidal neurons, excitatory connection, thalamocortical projection, corticothalamic projection neurons, corticospinal projection neurons, motor cortex

## Abstract

In spite of recent progress in brain sciences, the local circuit of the cerebral neocortex, including motor areas, still remains elusive. Morphological works on excitatory cortical circuitry from thalamocortical (TC) afferents to corticospinal neurons (CSNs) in motor-associated areas are reviewed here. First, TC axons of motor thalamic nuclei have been re-examined by the single-neuron labeling method. There are middle layer (ML)-targeting and layer (L) 1-preferring TC axon types in motor-associated areas, being analogous to core and matrix types, respectively, of Jones (1998) in sensory areas. However, the arborization of core-like motor TC axons spreads widely and disregards the columnar structure that is the basis of information processing in sensory areas, suggesting that motor areas adopt a different information-processing framework such as area-wide laminar organization. Second, L5 CSNs receive local excitatory inputs not only from L2/3 pyramidal neurons but also from ML spiny neurons, the latter directly processing cerebellar information of core-like TC neurons (TCNs). In contrast, basal ganglia information is targeted to apical dendrites of L2/3 and L5 pyramidal neurons through matrix TCNs. Third, L6 corticothalamic neurons (CTNs) are most densely innervated by ML spiny neurons located just above CTNs. Since CTNs receive only weak connections from L2/3 and L5 pyramidal neurons, the TC recurrent circuit composed of TCNs, ML spiny neurons and CTNs appears relatively independent of the results of processing in L2/3 and L5. It is proposed that two circuits sharing the same TC projection and ML neurons are embedded in the neocortex: one includes L2/3 and L5 neurons, processes afferent information in a feedforward way and sends the processed information to other cortical areas and subcortical regions; and the other circuit participates in a dynamical system of the TC recurrent circuit and may serve as the basis of autonomous activity of the neocortex.

## MOTOR-ASSOCIATED AREAS IN RODENTS

Motor-associated areas in the rodent cerebral cortex here include the primary motor (M1), secondary motor (M2), forelimb (FL), and hindlimb (HL) areas (Paxinos and Watson, 2007). Areas M1 and M2 correspond to lateral and medial agranular areas, respectively, of Donoghue and Wise (1982). Areas FL and HL have first been included in the primary somatosensory area (area S1; SmI neocortex of Welker, 1971), but later considered as mixed areas of motor and somatosensory information processing for the limbs. Although areas FL and HL are granular with developed layer (L) 4 and respond to somatosensory stimuli like area S1 for the face and trunk (Welker, 1971; Donoghue et al., 1979), area HL and the medial part of area FL have as low a threshold for intracortical microstimulation to evoke a motor response as area M1 (Hall and Lindholm, 1974; Donoghue and Wise, 1982; Sanderson et al., 1984; Neafsey et al., 1986; Tennant et al., 2011). A recent optogenetic stimulation technique with channelrhodopsin-2 expression in pyramidal neurons has supported the overlap of the M1 and somatosensory areas for the HL and FL (Ayling et al., 2009). Furthermore, when corticospinal projection neurons (CSNs) are labeled by injection of retrograde tracers into the corticospinal tract at the rat cervical spinal cord, many labeled neurons are continuously found in L5 from area M1 of the lateral agranular field to areas HL and FL of the lateral granular field (Wise and Jones, 1977; Leong, 1983; Miller, 1987; Killackey et al., 1989; Kaneko et al., 2000; Cho et al., 2004b; Tanaka et al., 2011a). Thus, area HL and the medial part of area FL are considered to have characteristics of motor areas, and, together with areas M1 and M2, treated as motor-associated areas in the present review.

Since areas M1 and M2 of rodents are called “agranular areas” as motor areas of higher mammals, these areas have generally been considered to lack L4. It is, however, often intriguing from the time of Krieg (1946) whether areas M1 and M2 in rodents have L4 or not. For example, Skoglund et al. (1997) reported the presence of L4 in area M1 by using the computerized analysis system based on their optical dissector method. Their conclusion was later supported by the presence of L4 in rat area M1 by using immunoreactivity for vesicular glutamate transporter 2 (VGluT2), which is a marker for thalamic afferents in the cerebral cortex (Fujiyama et al., 2001). The VGluT2-immunoreactive band in area M1 is continuous to that of area S1, and VGluT2 immunoreactivity in the band is as intense as that in L4 of area S1, although the band is thinner than L4 of area S1 (Cho et al., 2004a). However, in the present review, “the deepest part of L3 (L3d)” is conservatively used instead of “L4” in areas M1 and M2 to indicate the cortical layer receiving massive afferents from the thalamic nuclei, and “L2/3” is applied to superficial layers excluding this L3d to keep L2/3 of areas M1 and M2 homologous to L2/3 of areas HL and FL.

## INTRODUCTION OF LOCAL CIRCUIT ANALYSIS IN THE MOTOR-ASSOCIATED AREAS

The local excitatory connection of the rodent neocortex has been initially examined by the combination of intracellular recording and focal electrical stimulation (Connors et al., 1982; Chagnac-Amitai and Connors, 1989; Sutor and Hablitz, 1989; Silva et al., 1991; Hwa and Avoli, 1992). However, the results of the focal electrical stimulation in the neocortex are difficult to interpret, because it is unclear which components in the tissue are stimulated. Researchers may like to activate neuronal cell bodies and their local axon collaterals in the focal stimulation site, but afferent axons from thalamic nuclei and other cortical areas can also be activated. This uncertainty has been removed by the combined technique of intracellular recording and spike-triggered averaging (Thomson et al., 1988), or by the paired intracellular or whole-cell recording technique with intracellular stimulation (Thomson and West, 1993; Markram and Tsodyks, 1996; Buhl et al., 1997; Ohana and Sakmann, 1998; Galarreta and Hestrin, 1999). In the rodent neocortex including motor-associated areas, the synaptic connection between excitatory neurons has been examined extensively (Thomson and West, 1993; Deuchars et al., 1994; Thomson, 1997; Thomson et al., 2002; Bannister and Thomson, 2007). The paired recording technique is useful for examining the electrophysiological and pharmacological properties of monosynaptic connections between the excitatory neurons, and a high connectivity rate between neuronal groups, such as L4-to-L2/3 and L2/3-to-L5 connectivity rates (Thomson and Bannister, 1998; Thomson et al., 2002; Bannister and Thomson, 2007), suggests strong connections between the groups. However, the technique is usually unsuitable for quantitatively estimating connectivity between excitatory neuron groups because of sample selection biases. To remove the biases, Lefort et al. (2009) have quantified connectivity maps between excitatory neurons within a barrel column of mouse area S1 by randomly sampling a large number (2550) of excitatory neurons and testing 8895 possible synaptic connections within the column. Although this multiple whole-cell recording technique with random sampling is effective in mapping the local excitatory connections of the neocortex, no similar studies have been reported in the motor-associated areas yet.

Recently, another method for investigating cortical local connections has been developed by a combination of the whole-cell clamp recording and scanning laser photostimulation with caged glutamate in cortical slices (Dalva and Katz, 1994; Katz and Dalva, 1994). This photo-uncaging technique is useful for the selective stimulation of neuronal cell bodies, and has been applied not only to sensory cortical slices but also to motor cortical ones. For instance, in the mouse motor–frontal areas or vibrissal region of area M1, the photostimulation of L2/3 frequently evokes excitatory postsynaptic currents (EPSCs) in L5 pyramidal neurons (Weiler et al., 2008; Yu et al., 2008; Hooks et al., 2011). In addition, it has been reported that upper L5b CSNs and lower L5a crossed corticostriatal neurons, the latter of which send axons to the contralateral striatum, receive excitatory inputs from L2/3 neurons, whereas lower L5b CSNs accept inputs mainly from L5b neurons (Anderson et al., 2010). Further recently, the subcellular channelrhodopsin-2-assisted circuit mapping has been introduced (Petreanu et al., 2009). This optogenetic technique has revealed that neurons in area M1 send excitatory connections onto the apical dendrites of L2/3 and L5b pyramidal neurons and onto the basal dendrites of L5b neurons in the primary somatosensory area. These scanning laser photo-uncaging and optogenetic techniques are helpful in analyzing local or remote inputs to single cortical neurons.

There are only a few quantitative morphological analyses of local excitatory connections in the rodent neocortex. Using the electron-microscopic technique, Somogyi (1978) reported that excitatory asymmetric synapses with the intracortical axon collaterals of L4 pyramidal neurons were evenly found on the dendritic shafts of presumed interneurons and on the dendritic spines of excitatory spiny neurons in L4 of rat primary visual area (area V1). A similar result was reported in mouse area S1 by White and Hersch (1981). In contrast, the local collaterals of area M1-projecting pyramidal neurons in L3 of mouse area S1 preferred dendritic spines (~85%) within L3 and L5 of area S1 as their synaptic targets (Figures 3.1 and 7.1 in White, 1989). It is further interesting that most local axon collaterals (≥90%) of L5–L6 corticothalamic neurons (CTNs) in mouse area S1 terminated on the dendritic shafts of presumed interneurons within L4–L6 (White and Keller, 1987). Since only 37–46% of total asymmetric synapses were located on dendritic shafts in neuropil of L4–L5 of area S1 (Figure 7.1 in White, 1989), the local collaterals of CTNs clearly preferred the dendritic shafts of presumed interneurons as their targets. Although these electron-microscopic results suggest the presence of some specific connections in the intracortical circuitry of excitatory neurons, postsynaptic neuron groups are not fully identified except that they belong to spiny projection neurons or to non-spiny interneurons.

In our laboratory, several attempts have been made to find out a technique for breaking this limitation in the identification of postsynaptic neuron groups, and some results were obtained on the local circuit of the rat motor-associated areas. The method was basically composed of the specific retrograde or transgenic labeling approach and conventional intracellular staining technique: on one hand, the information-receiving sites (cell body and dendrites) of a functional group of cortical neurons were visualized by the Golgi stain-like retrograde labeling technique (Kaneko et al., 1996, 2000; Cho et al., 2004b; Tanaka et al., 2011a) or by the transgenic method for the expression of somatodendritic membrane-targeted green fluorescent protein (GFP; Tanaka et al., 2011b; Kameda et al., 2012); and, on the other hand, the local axonal arborization of single neurons was labeled by the sharp electrode intracellular (Kaneko et al., 2000; Cho et al., 2004b; Tanaka et al., 2011b) or whole-cell clamp recording technique (Tanaka et al., 2011a) with thick cortical slices. Subsequently, the local connection of single cortical neurons to the functional neuron group was investigated morphologically and quantitatively. This technique for detecting “one-to-group” connection is considered to work as a complementary method for the scanning laser photo-uncaging and optogenetic experiments, where the inputs of a neuron group to one neuron are investigated. In the present review, the previous morphological findings obtained by the “one-to-group” connection analysis are introduced and discussed with a focus on the local excitatory connections of the motor-associated areas. In addition, since the thalamocortical (TC) afferents are the starting point of information processing in motor-associated areas, this review first describes the recent progress in the study of cortical projection of single TC neurons (TCNs) in the motor thalamic nuclei.

## THALAMOCORTICAL INPUTS TO THE MOTOR-ASSOCIATED AREAS

**FIGURE 1:**
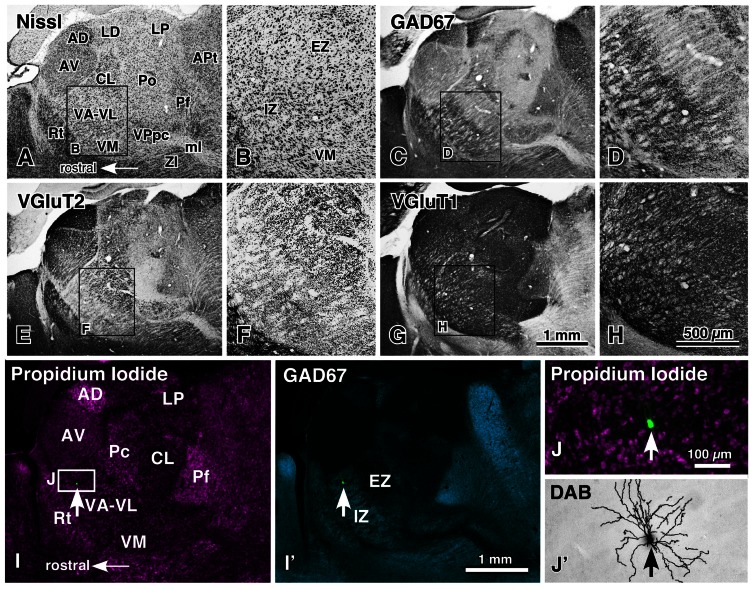
**Motor thalamic nuclei and single-neuron labeling with a viral vector expressing membrane-targeted GFP**. The motor thalamic complex VA–VL of rats is divided into two portions, IZ and EZ **(A,B)**. The rostroventrally located IZ receives abundant basal ganglia inputs that are large varicosities immunoreactive for GAD67 **(C,D)**, whereas the caudodorsally situated EZ admits cerebellar inputs consisting of many giant VGluT2-immunoreactive terminals **(E,F)**. Thus, the two portions are called inhibitory input-dominant zone (IZ) and excitatory subcortical input-dominant zone (EZ), respectively. In contrast, fine cortically derived VGluT1-immunoreactive axon terminals are distributed rather homogeneously not only in the VA–VL, but also in the entire thalamic nuclei **(G,H)**. When an appropriately diluted solution of viral vectors expressing palGFP is injected into the VA–VL, single neurons are labeled green by chance (arrows in **I,I′,J**), and visualized up to the tip of the dendrites by the immunoperoxidase staining **(J′)**. AD, anterodorsal nucleus; APt, anterior pretectal area; AV, anteroventral nucleus; CL, central lateral nucleus; LD, lateral dorsal nucleus; LP, lateral posterior nucleus; ml, medial lemniscus; Pc, paracentral nucleus; Pf, parafascicular nucleus; Po, posterior nucleus; Rt, thalamic reticular nucleus; VM, ventral medial nucleus; VPpc, parvocellular part of the ventral posterior nucleus. Modified with permission from Figure 1 of Kuramoto et al. (2011) and Figure 2 of Kuramoto et al. (2009). Scale bar in **(H)** applies to **(A–H)**, that in **(I′)** to **(I,I′)**, and that in **(J)** to **(J,J′)**.

The ventral anterior and ventral lateral thalamic nuclear complex (VA–VL) is the motor thalamic nuclei, receiving cerebellar and basal ganglia afferents and sending projections to motor-associated cortical areas. The VA–VL is divided into two portions (**Figures 1A–H**; Kuramoto et al., 2009, 2011): the rostroventrally located inhibitory input-dominant zone (IZ) and caudodorsally situated excitatory subcortical input-dominant zone (EZ). The IZ of the VA–VL contains large axon terminals immunoreactive for GABA-synthesizing enzyme (glutamic acid decarboxylase of 67 kDa, GAD67), whereas the EZ is filled with giant axon terminals with VGluT2 immunoreactivity. These GAD67- and VGluT2-immunoreactive terminals have been extensively reduced by a large lesion in the substantia nigra and deep cerebellar nuclei, respectively (Kuramoto et al., 2011). This indicates that the IZ is principally innervated by the basal ganglia inhibitory afferents, whereas the EZ is mainly driven by the cerebellar excitatory afferents.

**FIGURE 2:**
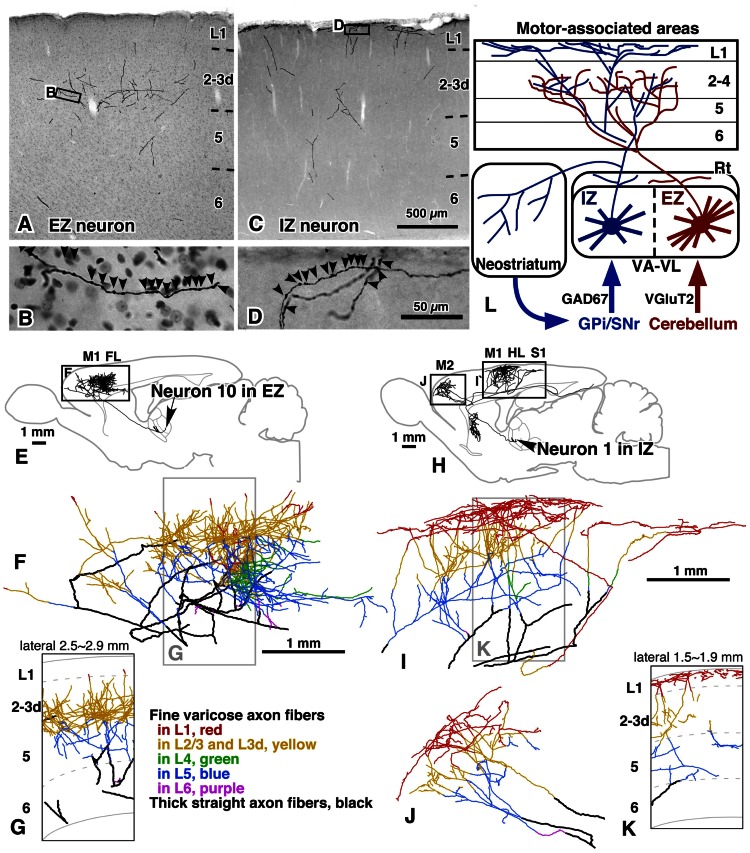
**Axonal arborization of EZ and IZ neurons in rat cerebral cortex and striatum**. The axons of both EZ and IZ neurons were distributed widely in motor-associated areas; the axon fibers of EZ neurons preferred middle cortical layers, L3–L5, as their target layers **(A,B,E–G)**, whereas those of IZ neurons were mainly (>50%) distributed in L1 **(C,D,H–K)**. In addition, IZ neurons but not EZ neurons sent a considerable amount of axon collaterals to the striatum. The results are schematically summarized in **(L)**. Modified with permission from Figures 5,6, and 9 of Kuramoto et al. (2009). Scale bar in **(C)** applies to **(A,C)**; that in **(D)** to **(B,D)**; and those in **(F,I)** to **(F,G,I–K)**.

The whole axonal arborization of single IZ and EZ neurons was further investigated, using a viral vector expressing membrane-targeted GFP (palGFP). By injection of appropriately diluted solution of the viral vector into the VA–VL, single-neuron labeling of IZ or EZ neurons was obtained by chance (**Figures 1I–J′**). Because of the strong expression of palGFP in the infected neuron, the whole axonal arborization of single neurons was visualized up to the end of the axons (**Figures 2A–D**). When the axonal arborization was reconstructed, the following differences between IZ and EZ neurons were noticed (**Figures 2E–K**):

(1) The cortical axons of IZ neurons preferred L1 of motor-associated areas, 54.0 ± 7.3% of intracortical axon boutons being distributed in L1. In contrast, only 5.8 ± 5.1% of intracortical boutons of EZ neurons were found in L1, and mainly distributed in middle layers (MLs; L3–L4).
(2) Almost no EZ neurons sent axon collaterals to the striatum, whereas all IZ neurons projected a considerable amount of collaterals to the striatum.
(3) The cortical axonal arborization of IZ neurons was very wide in areas M1, M2, HL, FL and S1. The arborization of EZ neurons was also widespread, but narrower than that of IZ neurons.
(4) The dendritic arborization of EZ neurons was denser than that of IZ neurons.

**FIGURE 3:**
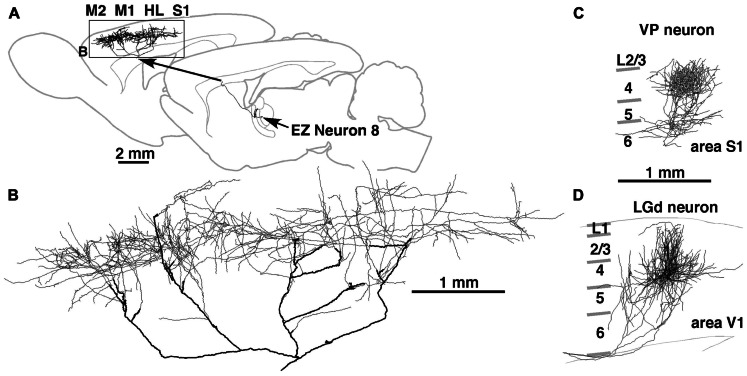
**Axonal arborization of an EZ neuron in comparison with that of primary sensory thalamic neurons in rats**. The axons of EZ neurons were distributed very widely, often in the cortical area spanning more than 5 mm **(A,B)**. This distribution is in sharp contrast to those of sensory thalamic neurons, such as neurons in the VP and dorsal lateral geniculate nucleus (LGd). The axonal arborization of VP and LGd neurons was concentrated to the middle layers of a cortical region with the size of a single column **(C,D)**. Modified with permission from Figure 8 of Kuramoto et al. (2009) **(A,B)** and Figure 3 of Furuta et al. (2011) **(C)**. The axonal arborization of a single LGd neuron in area V1 **(D)** was labeled and illustrated with the same method as that in Kuramoto et al. (2009) by Nakamura and Kaneko.

These results are partly compatible with the concept of “core” and “matrix” projections of TCNs, proposed mainly in the sensory thalamic neurons by Jones (for review, see Jones, 1998, 2001). In the monkey and cat thalamic nuclei, core-type neurons are immunopositive for parvalbumin and mainly form spatially restrictive projection to cortical MLs with the size of a functional column, whereas the matrix-type neurons are positive for calbindin D28k and send their axons preferentially and widely to L1. In the concept of Jones, it is the most important point that matrix-type neurons are distributed throughout the thalamic nuclei. In addition to the L1-preferring wide arborization of IZ axons, the IZ was filled with calbindin-immunoreactive cell bodies (Kuramoto et al., 2009). Thus, IZ neurons are considered to fulfill the definition of matrix-type neurons. On the other hand, because no thalamic neurons are positive for parvalbumin in rodents, only the spatially restrictive, columnar projection to the middle cortical layers can be used to identify core-type neurons in rodent TCNs. Actually, this columnar projection of TCNs to the MLs has been reported in primary sensory areas of rats (Furuta et al., 2011) as observed in those of monkeys and cats. However, the cortical axons of EZ neurons were widely, though not evenly, distributed in the motor-associated areas (**Figures 3A,B**), although their main target layers were L3d in areas M1 and M2 and L4 in areas FL and HL as those of core-type somatosensory and visual relay neurons (**Figures 3C,D**). Thus, EZ neurons are tentatively named “core-like” neurons here. This difference in axonal arborization between sensory core-type and motor core-like TCNs suggests that motor-associated areas adopt a different information-processing framework from that of sensory areas. In other words, the motor-associated areas might apply “non-columnar,” area-wide information processing for motor control. Furthermore, because the axonal arborizations of both EZ and IZ neurons were widely distributed, single pyramidal neurons with developed apical dendrites in the motor-associated areas are likely to receive and integrate two kinds of motor information: one from the basal ganglia to the apical dendrites of pyramidal neurons, and the other from the cerebellum to their basal dendrites.

## LOCAL INPUTS TO CORTICOSPINAL NEURONS

**FIGURE 4:**
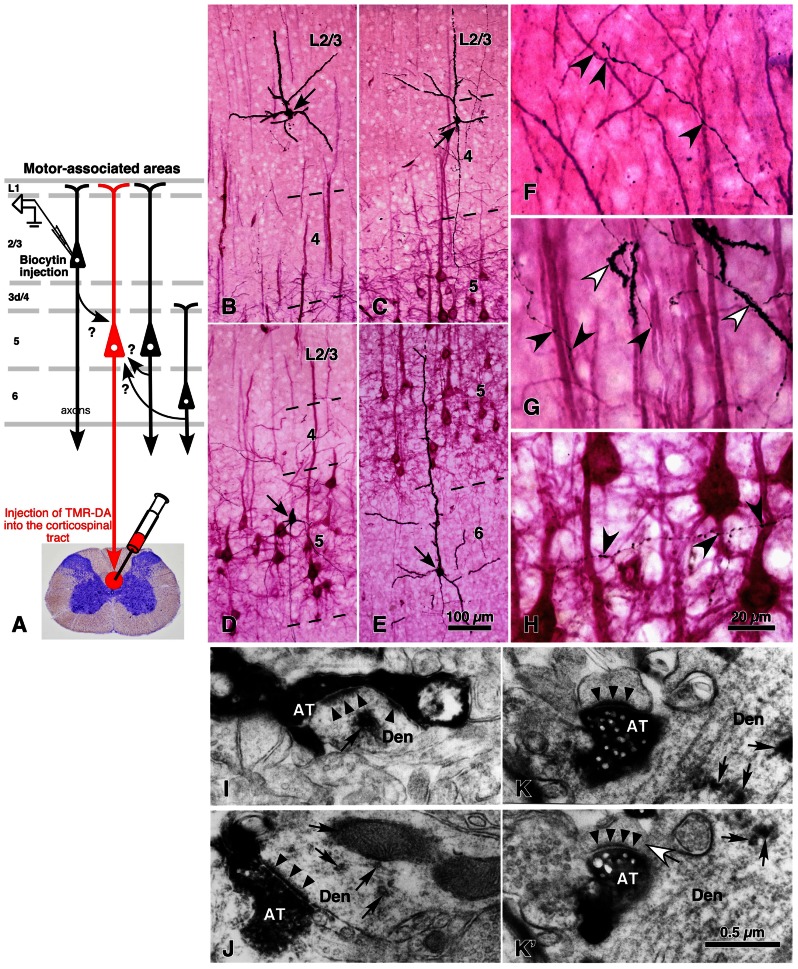
**Local excitatory inputs to CSNs in rat cerebral cortex**. The dendrites of CSNs in the motor-associated areas were retrogradely labeled in a Golgi stain-like manner by the injection of tetramethylrhodamine-dextran amine (TMR-DA) into the corticospinal tract in the cervical cord with an acidic vehicle (**A**; Kaneko et al., 1996). In 500-μm-thick cortical slices containing labeled CSNs, pyramidal/spiny neurons were recorded and labeled intracellularly at each cortical layer (arrows in **B–E**). The axodendritic appositions between black-labeled axon varicosities and red-visualized dendrites (black arrowheads in **F–H**) were quantitatively analyzed. In some samples, the appositions were confirmed to make synaptic contacts in electron-microscopic images **(I–K′)**, where black arrowheads and arrows indicated the postsynaptic densities and immunoreaction products for TMR-DA, respectively. Figure **(K)** is the image next to **(K′)**, in which a white arrow points to the spine neck connecting the unlabeled spine to the TMR-DA-labeled dendrite. **(B–K′)** Modified with permission from Figures 1 and 8 of Cho et al. (2004b). AT, intracellularly labeled axon terminals; Den, TMR-DA-labeled dendritic profiles. Scale bar in **(E)** applies to **(B–E)**, that in **(H)** to **(F–H)**, and that in **(K′)** to **(I–K′)**.

**FIGURE 5:**
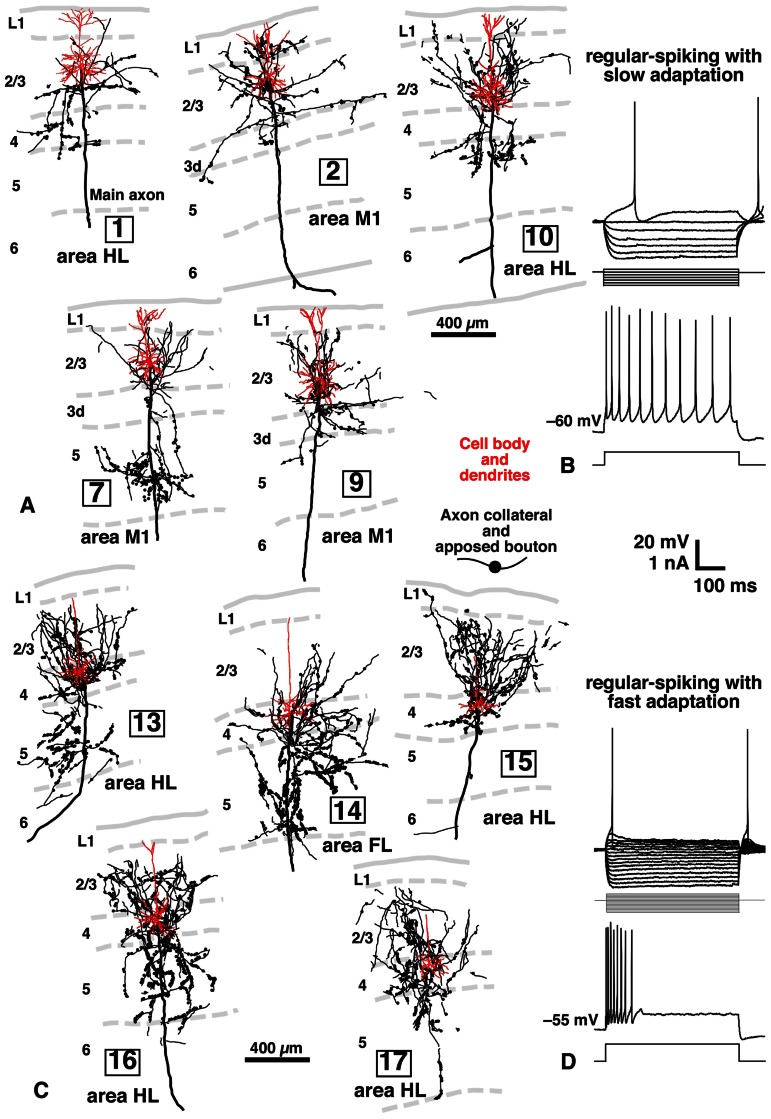
**Inputs of L2/3 pyramidal and L3d and L4 spiny neurons to CSNs, and electrical properties of L2/3 and L3d/L4 neurons in rat brain**. Many axon boutons of L2/3 pyramidal neurons were in close apposition to the apical or basal dendrites of CTNs **(A)**. However, unexpectedly, the axons of L3d/L4 star-pyramidal neurons formed more appositions with the dendrites of CTNs **(C)** than those of L2/3 pyramidal neurons. The two groups of excitatory neurons were different in electrical properties: L2/3 pyramidal neurons showed regular-spiking responses with slow adaptation **(B)**, whereas L3d/L4 star-pyramidal neurons exhibited regular-spiking ones with fast adaptation **(D)**, when a long depolarizing current pulse was injected. Modified with permission from Figures 2–4 of Cho et al. (2004b) and Figures 4 and 5 of Cho et al. (2004a).

In contrast to the previous section on thalamic inputs, output neurons of motor-associated areas are a main subject in this section. The group of CSNs can be retrogradely labeled up to the tip of the dendritic processes by injection of tetramethylrhodamine-dextran amine into the corticospinal tract with an acidic vehicle (**Figure 4A**; Kaneko et al., 1996). With this technique, more than 45% of L5 neurons were efficiently labeled (red stained neurons in **Figures 4B–H**; Kaneko et al., 2000; Cho et al., 2004b), and it was assumed that the vast majority of CSNs were visualized in motor-associated areas (see Discussion in Cho et al., 2004b). In 500-μm-thick cortical slices containing retrogradely labeled L5 CSNs, single pyramidal/spiny neurons that were located in each cortical layer were labeled intracellularly for the “one-to-group” connection analysis. The appositions formed between the local axon collaterals of the intracellularly labeled pyramidal neurons and the dendrites of CSNs were traced as shown in **Figure 5**. In a different set of experiments, about 60–77% of appositions were electron-microscopically confirmed to make axodendritic synaptic contacts of asymmetric type mainly on dendritic spines (**Figures 4I–K′** and **8O–R**; Cho et al., 2004b; Tanaka et al., 2011b), suggesting that the number of appositions could be applied as a quantitative indicator of synaptic connections.

**FIGURE 6:**
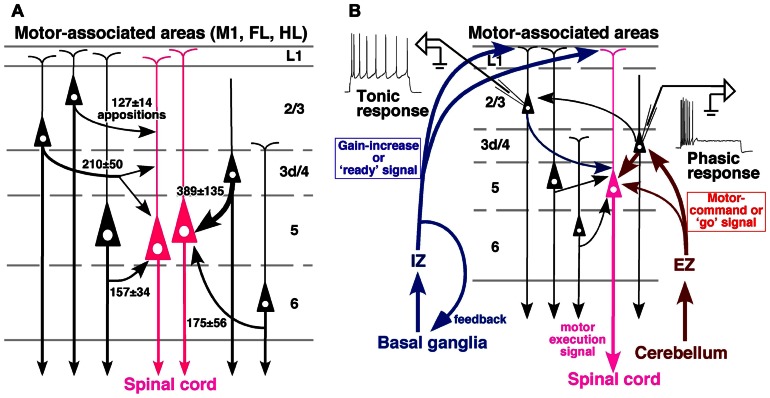
**Summary of local excitatory inputs to CSNs and hypothesized circuit for motor control**. **(A)** Although lower L2/3 pyramidal neurons send a considerable number of apposed boutons to CSNs, the strongest inputs are derived from L3d/L4 star-pyramidal neurons. The thickness of a curved arrow indicates the relative intensity of the projection. **(B)** A conceivable cortical circuit for motor control, which is composed of thalamic afferents and local connections to CSNs. Modified with permission from Figure 9 of Cho et al. (2004b) and Figure 9 of Kuramoto et al. (2009).

As summarized in **Figure 6A**, L5 CSNs received local inputs from all the cortical layers with some differences in connectional weight (the number of appositions/presynaptic neuron). The pyramidal neurons in the upper half of L2/3 (upper L2/3) sent the least number of appositions to CSNs (neurons 1 and 2 in **Figure 5A**), but those in the lower half of L2/3 (lower L2/3) projected densely to CSNs (neurons 7, 10, and 12). This result is consistent with the previous observation in the cat motor cortex (Kaneko et al., 1994a,b); pyramidal neurons receiving monosynaptic inputs from area 2 were located mainly in lower L2/3, made two axon collateral bushes in L2/3 and L5, and projected densely to L5 pyramidal neurons including Betz cells, whereas pyramidal neurons accepting polysynaptic inputs alone were situated more superficially in L2/3, formed a single collateral bush in L2/3 and sent much fewer axons to L5 pyramidal neurons.

Furthermore, it was an unexpected and interesting result that L3d and L4 star-pyramidal neurons, which had an apical dendrite without tufts, were the most abundant source of inputs to CSNs among the pyramidal neurons examined (**Figures 5B** and **6A**). In L3d of area M1 and L4 of areas HL and FL, about 2/3 of spiny cells were star-pyramidal neurons, and the remaining 1/3 were pyramidal neurons (Cho et al., 2004a). The lack or poverty of apical tufts suggests that these star-pyramidal neurons would not receive the matrix-type IZ afferents that were discussed in the previous section to transmit the basal ganglia information preferentially to L1 (**Figure 6B**). It was further interesting that all these L3d and L4 star-pyramidal neurons showed regular-spiking responses with fast adaptation to current pulse injections (**Figure 5D**; Cho et al., 2004a). The phasic responses of L3d and L4 star-pyramidal neurons suggest that these neurons serve as a kind of high-pass/low-cut filter to the core-like EZ afferents, which mainly convey cerebellar information (**Figure 6B**). In contrast, L2/3 neurons consistently displayed regular-spiking responses with slow adaptation, which resulted in a tonic activity during current pulse injections (**Figure 5B**).

These results suggest the following local circuits in motor-associated areas (**Figure 6B**):

(1) Basal ganglia information directly enters the apical dendrites not only of L2/3 pyramidal neurons but also of L5 CSNs through IZ neurons of the VA–VL. Because L1-preferring TC afferents are associated with the cortical activity prior to the motor execution (for review, see Roland, 2002) and modulate the gain of pyramidal cell response (Larkum et al., 2004), the basal ganglia system may give motor preparatory information to CSNs through its disinhibitory mechanism on IZ neurons.
(2) L2/3 neurons in rodent area M1 further receive information from the other cortical areas such as the somatosensory cortex by corticocortical connection (Akers and Killackey, 1978; Welker et al., 1988; Hoffer et al., 2003) as well as cerebellar information *via* L3d and L4 neurons. Because movement-related potentials such as the readiness potential (Bereitschaftspotential; Kornhuber and Deecke, 1965), which is the cortical activity preceding the movement, are known to occur mainly in L2/3 (for review, see Colebatch, 2007), the tonic firing property of L2/3 pyramidal neurons may be helpful in developing a preparatory activity of CSNs. In addition, the tonic activity of L2/3 pyramidal neurons may be useful in maintaining the activity of CSNs during the motor execution through L2/3-to-L5 excitatory connection.
(3) On the other hand, cerebellar motor command is mainly transferred to L3d and L4 star-pyramidal neurons through EZ neurons of the VA–VL, and sent to L5 CSNs as well as to L2/3 neurons. Since L3d and L4 star-pyramidal neurons show the characteristics of a high-pass filter, timing information within the cerebellar command or “go” signal may be conveyed to CSNs *via* this connection. It is thus presumed that, when CSNs are prepared for a motion by the “ready” signal from the basal ganglia or other cortical areas, CSNs are easily activated in the exact timing by the cerebellar “go” signal and discharge a motor execution signal to the spinal cord (**Figure 6B**).

## LOCAL INPUTS TO CORTICOTHALAMIC NEURONS

**FIGURE 7:**
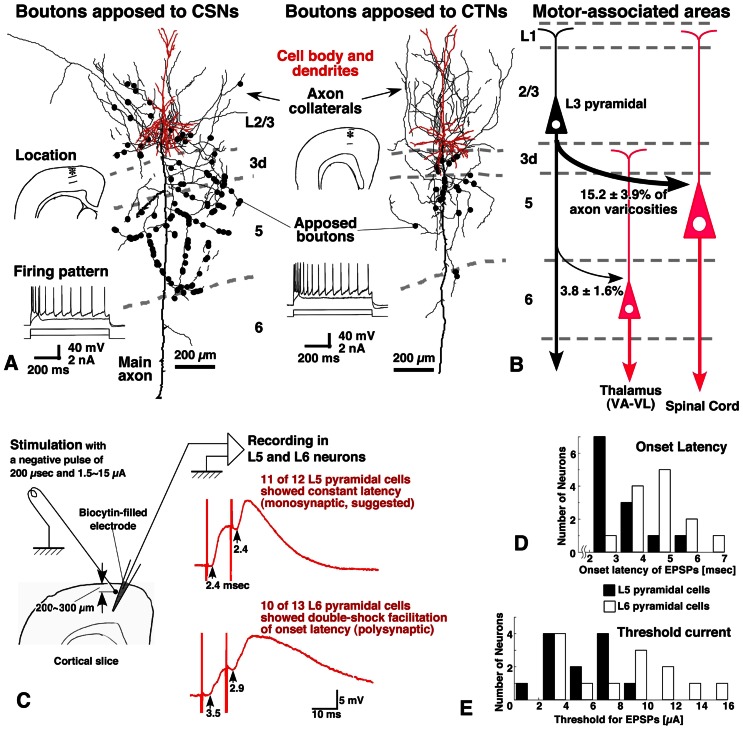
**Inputs of L3 pyramidal neurons to CSNs and CTNs in rat motor-associated areas**. The morphological experiments were performed in a similar way to that illustrated in **Figure 4A**, except that the injection was made not only into the corticospinal tract but into the VA–VL. L3 pyramidal neurons preferentially sent apposed boutons to CSNs, but much fewer (about 1/4) to CTNs **(A,B)**. When the superficial layers of motor-associated areas were stimulated electrically (left figure in **C**), EPSPs were evoked with shorter latencies and lower thresholds in L5 pyramidal neurons than in L6 pyramidal neurons **(D,E)**. Furthermore, in double-shock stimulation experiments, EPSPs in 11 of 12 L5 pyramidal neurons showed a constant latency, being a sign of monosynaptic inputs, whereas those in L6 pyramidal neurons displayed double-shock facilitation of onset latencies, indicating that the connection was polysynaptic **(C)**. These morphological and electrophysiological findings suggest that L6 pyramidal neurons do not receive strong monosynaptic inputs from L3 pyramidal neurons. **(A,C–E)** Modified with permission from Figures 4, 6, and 7 of Kaneko et al. (2000).

Corticothalamic neurons in motor-associated areas were mainly located in L6 and sent their axons massively to the VA–VL of the thalamus. In comparison to CSNs, CTNs received much less information from L2/3 pyramidal neurons (Kaneko et al., 2000). Actually, single L2/3 pyramidal neurons sent much fewer (~1/4) axon varicosities to CTNs than to CSNs by the quantitative “one-to-group” connection analysis as described above (**Figures 7A,B**). This was confirmed electrophysiologically (Kaneko et al., 2000); the electrical stimulation in L2/3 of motor-associated areas produced excitatory postsynaptic potentials (EPSPs) with a short and constant latency in L5 pyramidal neurons (**Figures 7C,D**), suggesting a monosynaptic connection from L2/3 excitatory neurons to L5 pyramidal neurons. This result is supported by recent experiments of photo-uncaging stimulation, in which L5b pyramidal neurons or CSNs in the mouse motor area received excitatory monosynaptic inputs from L2/3 neurons (Anderson et al., 2010; Hooks et al., 2011). In contrast, EPSPs observed in L6 pyramidal neurons showed longer latencies, and higher stimulation currents were needed to evoke EPSPs (**Figures 7D,E**). These EPSPs often exhibited double-shock facilitation of onset latencies (**Figure 7C**), and were suppressed by blocking *N*-methyl-D-aspartate receptors (Kaneko et al., 2000), indicating the polysynaptic nature of the EPSPs. These morphological and electrophysiological results suggest that L6 CTNs in motor-associated areas are relatively independent of the information that is processed in L2/3.

**FIGURE 8:**
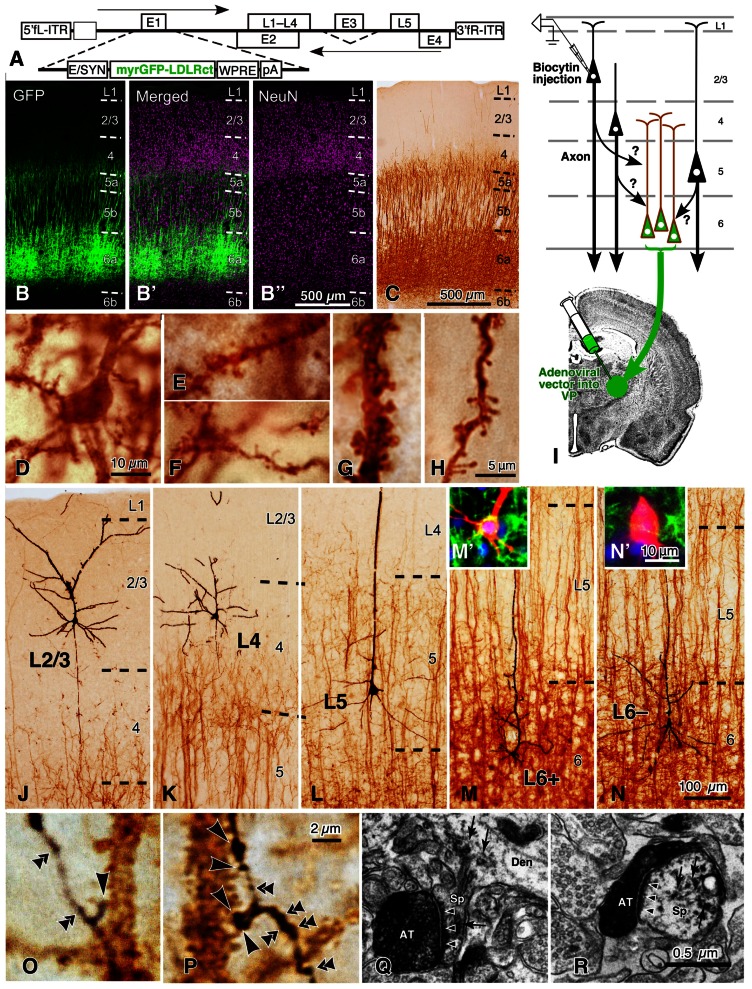
**Golgi stain-like labeling of CTNs with a viral vector and intracellular staining of pyramidal neurons in rat areas S1, FL, and HL**. When high titers of adenoviral vectors expressing myrGFP-LDLRct (**A**; Kameda et al., 2008) were injected into the VP with 0.6 M NaCl, many L6 pyramidal neurons were retrogradely infected in the somatosensory motor area **(B–B″)**. After the brown immunoperoxidase staining with anti-GFP antibody and diaminobenzidine (DAB; **C**), the cell body **(D)**, basal dendrites **(E,F)**, and apical dendrites **(G,H)** of CTNs were fully visualized. Note that even fine spines were visualized effectively. In 500-μm-thick cortical slices, single spiny neurons were labeled intracellularly **(I)** and visualized black **(J–N)** by the peroxidase method with DAB and nickel. In L6, retrogradely labeled **(M′)** and unlabeled neurons **(N′)** were indicated by L6^+^ and L6^–^ pyramidal neurons, respectively. Most L6^–^ neurons were considered to belong to corticocortical projection neurons, because their apical dendrites were short and the basal dendrites were abundant as reported previously (Zhang and Deschênes, 1997). In contrast, L6^+^ CTNs were taller and more slender than L6^–^ neurons. **(O,P)** It was examined whether each axon bouton of the intracortical collaterals was in close apposition to the retrogradely labeled CTN dendritic spines (large arrowheads) or not (double arrowheads). **(Q,R)** In addition, 77% of those appositions were electron-microscopically confirmed to form asymmetric synaptic contacts with the labeled spines (small arrowheads). The reaction products of retrograde labeling are indicated by small arrows. AT, labeled axon terminals; Den, dendritic profile; Sp, spine. Modified from Figures 1, 2, and 4 of Tanaka et al. (2011b). Scale bar in **(B″)** applies to **(B–B″)**, that in **(H)** to **(E–H)**, that in **(N)** to **(J–N)**, that in **(N′)** to **(M′,N′)**, that in **(P)** to **(O,P)**, and that in **(R)** to **(Q,R)**.

Subsequently, a recent morphological analysis on the local excitatory inputs to CTNs is introduced here, although the analysis was performed in sensory areas (area S1, FL, and HL; Tanaka et al., 2011b). For the analysis, an adenoviral vector expressing somatodendritic membrane-targeted GFP (myrGFP-LDLRct; **Figure 8A**) was developed. After injection of a high-titer vector solution into the ventral posterior thalamic nuclei (VP) at a high-salt condition, CTNs were retrogradely infected (**Figures 8B–C**), and all their somatodendritic structures including thin portions and spines of the dendrites were visualized clearly (**Figures 8D–H**). About 60% of L6 neurons in the VP-projecting region of sensory areas were labeled with this technique, and the vast majority of CTNs were considered to be visualized in the region, because the labeling efficiency was saturated even by injection of a higher concentration of the vector (Tanaka et al., 2011b). For the “one-to-group” connection analysis of inputs to CTNs (**Figure 8I**), cortical slices containing many myrGFP-LDLRct-expressing CTNs were used; single pyramidal neurons in each cortical layer and their axon fibers were visualized black, and the dendrites of CTNs were stained brown (**Figures 8J–N**). The axon boutons of black axon fibers were frequently apposed to brown dendritic spines (**Figures 8O,P**), and most of them were revealed to make axospinous synaptic contacts under the electron-microscope (**Figures 8Q,R**).

**FIGURE 9:**
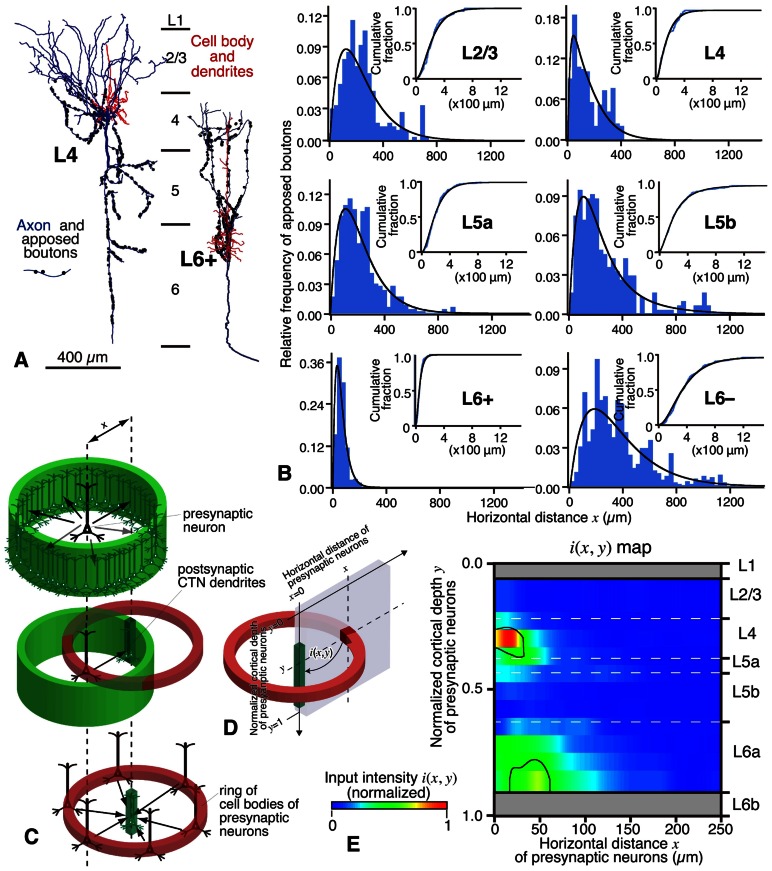
**Local excitatory inputs to VP-projecting CTNs in rat areas S1, HL, and FL**. **(A)** Single L4 star-pyramidal and L6^+^ pyramidal neurons sent many apposed boutons to CTNs. **(B)** The horizontal distribution (fitted with a gamma distribution) of the apposed boutons of representative neurons were different from layer to layer. L4 spiny and L6^+^ pyramidal neurons sent apposed boutons to CTN dendrites that were located in a narrow range, whereas L5 and L6^–^ neurons projected them to CTN dendrites that spread horizontally. **(C,D)** From the original data in **(B)**, the number of apposed boutons arising from an average presynaptic neuron as a function of horizontal distance *x* is obtained (**C**, top) under the assumption that cortical excitatory neurons sending axons to CTN dendrites are distributed homogeneously in horizontal directions at a given depth (*y*). When the presynaptic neuron sends a certain amount of apposed boutons to postsynaptic CTN dendrites in a given unit volume (slender dense green square prism in **C**, middle), the CTN dendrites are expected to receive the same amount of projections from each neuron located in all directions at the same distance from them (**C**, bottom). Therefore, as shown in **(D)**, a section can be cut out to make a two-dimensional input map; in other words, one can obtain input intensity map *i*(*x*, *y*), which is the density of axon boutons derived from presynaptic neurons within a red cube located at horizontal distance *x* and normalized cortical depth *y* and closely apposed to the postsynaptic CTN dendrites within the green square prism. In this estimate, the number of neurons in the cube located at (*x*, *y*) is calculated from the density of presynaptic VGluT1 mRNA-expressing neurons at depth *y*. **(E)** Input intensity map *i*(*x*, *y*). From the viewpoint of CTN dendrites in a unit prism, L4 spiny neurons are the most abundant source of local excitatory inputs. The regions encircled by black borders in **(E)** show significantly high *i*(*x*, *y*) (> mean + 2SD). For more detail, see Tanaka et al. (2011b). Modified with permission from Figures 5–8 of Tanaka et al. (2011b).

The results of the local excitatory inputs to CTNs in sensory areas are summarized in **Figure 9**. **Figure 9A** exemplifies the distribution of boutons closely apposed to CTNs along the local axon collaterals of an L4 star-pyramidal neuron and a retrogradely labeled L6 CTN (L6^+^ neuron; **Figure 8M′**). The local inputs of single excitatory neurons to the CTN group were in the following order (from the most abundant to the least): retrogradely unlabeled, presumably corticocortical L6 neurons (L6^–^ neurons; **Figure 8N′**), mean ± SD of the number of apposed boutons/presynaptic neuron = 953 ± 500 (25% of total axon boutons); L6^+^ pyramidal neurons, 612 ± 223 (35%); L5a pyramidal neurons, 529 ± 148 (10%); L5b pyramidal neurons, 374 ± 142 (22%); L4 spiny neurons, 327 ± 164 (6%); and L2/3 pyramidal neurons, 167 ± 115 (3%). The L2/3-to-CTN connection was thus weakest of the local excitatory connections to CTNs, being consistent with the previous results in motor-associated areas (Kaneko et al., 2000). Therefore, L5 pyramidal neurons and L4 spiny neurons, including spiny stellate, star-pyramidal and pyramidal neurons, were important sources of translaminar excitatory inputs to CTNs in terms of the number of apposed boutons/presynaptic neuron, although the local connection within L6 was most abundant. It is noticeable that single L6^+^ CTNs sent 35% of axon boutons to the CTN group, because this result appears contradictory to the previous finding that the local axon collaterals of CTNs principally targeted interneurons in mouse area S1 (White and Keller, 1987). This inconsistency is unlikely to be due to a species difference, as recent paired recording studies showed that CTN-to-L6 pyramid connectivity rate (1/75) was much lower than CTN-to-L6 interneuron connectivity rate (1/4) in rat area S1 and area V1 (Mercer et al., 2005; West et al., 2006). On the other hand, recent scanning laser photostimulation studies revealed that L6 CTNs or presumed CTNs preferentially received excitatory inputs from surrounding L6 neurons in rat area V1 (Zarrinpar and Callaway, 2006) and in mouse primary auditory area (Llano and Sherman, 2009), partly supporting the results of Tanaka et al. (2011b). Regardless, the postsynaptic components of L6 CTN axon collaterals should be investigated further.

In **Figure 9B**, it is noticed that, when the horizontal spread of these local connections was examined, L4 and L6^+^ neurons formed appositions with the CTN group in a narrower range than L2/3, L5, or L6^–^ pyramidal neurons (Tanaka et al., 2011b), suggesting that the spatial organization is of crucial importance to the understanding of local inputs to CTNs. In addition, to compare these spatial data with the maps observed in previous scanning laser photostimulation studies on excitatory inputs to L6 pyramidal neurons (Zarrinpar and Callaway, 2006; Llano and Sherman, 2009; Hooks et al., 2011), we tried to constitute an input map to a CTN, actually CTN dendrites in a unit volume, using the spatial information of the experimental data. When the inputs to CTNs are reconstructed from the viewpoint of a CTN, the following assumptions are made: (1) the density of CTN dendrites is constant in the horizontal direction at a given cortical depth (*y*), (2) the distribution of cell bodies of various pyramidal/spiny neurons is also horizontally constant at given depth *y*, and (3) as a group, pyramidal/spiny neurons at given depth *y* deliver their apposed boutons to CTNs isotropically but as a function of horizontal distance *x*. From the original data of the experiments (**Figure 9B**), one can obtain an input intensity map *i*(*x*, *y*), which is the density of boutons derived from presynaptic neurons within a cube located at horizontal distance *x* and normalized cortical depth *y* and closely apposed to the postsynaptic CTN dendrites within the green square prism (**Figures 9C,D**; for detail, see the legend of **Figure 9**). As a result, the two-dimensional map *i*(*x*, *y*) in **Figure 9E** reveals that the highest *i*(*x*, *y*) is located at L4 and the second highest is found at L6a. Thus, L4 and L6 pyramidal/spiny neurons are important local sources of inputs to CTNs, and at least a portion of L4 neurons have a strong impact on the CTNs that are located in a narrow region (≤40 μm) underneath these L4 neurons. This result is relatively compatible with the results of scanning laser photostimulation studies; presumed CTNs in L6 or L6 pyramidal neurons received significant, if not strong, excitatory inputs from L4 in rat area V1 (Zarrinpar and Callaway, 2006) or in mouse area S1 (Hooks et al., 2011), respectively, although paired whole-cell recording experiments did not detect a high connectivity rate of L4-to-L6 connection (Lefort et al., 2009). Finally, this strong L4-to-CTN connection appears to be formed by the descending axon collaterals of L4 spiny neurons (**Figure 9A**, left).

## LOCAL CIRCUITS IN MOTOR-ASSOCIATED AREAS AND DISCUSSION

**FIGURE 10:**
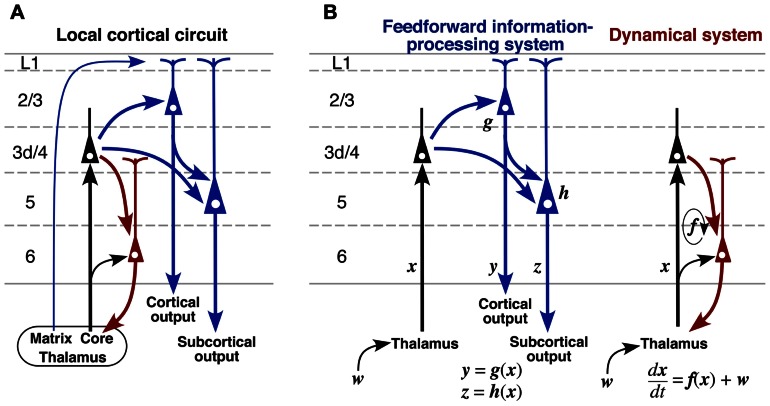
**A proposed model of cortical excitatory circuitry**. **(A)** L3d and L4 spiny neurons receive abundant core-type or core-like thalamic inputs, whereas L2/3 and L5 pyramidal neurons accept matrix-type inputs on their apical tufts. L2/3 pyramidal and L3d/L4 spiny neurons send axons to L5 pyramidal neurons, and L2/3 and L5 pyramidal neurons project to other cortical areas and subcortical regions, respectively (blue circuit). In contrast, L6 CTNs receive thalamic inputs directly and indirectly *via* L3d/L4 neurons, and then send their activity back to the thalamic neurons (red circuit). **(B)** It is likely that these two circuits are embedded in the cortical microcircuit with relative independence. The blue circuit may behave like a feedforward information-processing system, whereas the red one may serve as a dynamical system because of its recurrent nature. ***w***, subcortical or cortical “driver” afferent vector; ***x***, state vector of thalamic neurons; ***y***, output vector of L2/3 pyramidal neurons; ***z***, output vector of L5 pyramidal neurons.

A scheme shown in **Figure 10A** summarizes the main local excitatory connections in the cerebral cortex including motor-associated areas. When minor neuronal populations, such as L6 corticocortical neurons, and weak connections are omitted, the excitatory connections may be described in the following way:

(1) The cortex receives two kinds of TC afferents. The core-type or core-like TC projection mainly targets L3d and L4 spiny neurons and partially L6 pyramidal neurons. The latter may directly receive the TC projection because the apical dendritic tufts of CTNs are densely distributed in L4–L5a (**Figure 8C**) and partly because the core-type projection sends some axon collaterals to L6 (**Figure 3C**). In contrast, the matrix-type projection targets the apical dendritic tufts of L2/3 and L5 pyramidal neurons as discussed in Section “Thalamocortical Inputs to the Motor-associated Areas.”
(2) L2/3 pyramidal neurons may receive dense inputs from L3d and L4 neurons, which send dense axonal arborization to L2/3 as shown in motor-associated areas (**Figure 5C**) as well as in sensory areas (Figure 3 of Tanaka et al., 2011b). The presence of this dense connection is supported in rat neocortex by the paired electrical recording experiments revealing a relatively high connectivity rate in the L4-to-L2/3 connection (Thomson et al., 2002; Bannister and Thomson, 2007). In addition, the dense L4-to-L2/3 connection is constantly shown by scanning laser photostimulation studies in rodent sensory areas (Shepherd and Svoboda, 2005; Shepherd et al., 2005; Hooks et al., 2011). A similar dense connection to L2/3 of the motor areas is originated from the border region between L3 and L5, which might contain L3d (Wood et al., 2009; Wood and Shepherd, 2010). After the local information processing, L2/3 pyramidal neurons are well known to project to other cortical areas (for review, see Jones, 1984).
(3) L5 pyramidal neurons including CSNs receive massive inputs from L3d and L4 spiny neurons and less massive inputs from L2/3 neurons (**Figure 6A**), and send axons to subcortical regions including the spinal cord. The latter L2/3-to-L5 connection is supported in motor areas by scanning laser photostimulation studies (Weiler et al., 2008; Anderson et al., 2010; Hooks et al., 2011) and paired electrical recording experiments (Thomson and Bannister, 1998; Thomson et al., 2002).
(4) Corticothalamic neurons collect dense inputs from L4 spiny neurons, but rarely receive input connections from L2/3 or L5 (**Figure 9E**). The key point of this local connection scheme (**Figure 10A**) is the relative independence of CTNs from the information processing performed by L2/3 and L5 neurons, which are indicated with blue color in the scheme. This observation is supported by the laser scanning photostimulation studies revealing that L6 neurons receive very few, if any, inputs from L2/3 and L5 not only in area S1 (Zarrinpar and Callaway, 2006; Llano and Sherman, 2009; Hooks et al., 2011) but also in area M1 (Hooks et al., 2011).

Although the scheme in **Figure 10A** is commonly applicable to many cortical areas, a large difference in TC afferents between sensory and motor areas has to be emphasized here. The core-type afferents to the primary sensory areas basically show columnar organization as well as laminar arrangement (**Figures 3C,D**), and the afferent information is processed within a functional column at least in its initial step. However, in the case of motor thalamic neurons (**Figures 2** and **3**), the information of a TCN is delivered to area-wide cortical regions. This area-wide distribution of core-like motor thalamic afferents may be relevant to the fact that the motor information is already processed in the cerebellar cortex, although the relationship of cerebellar information processing to cerebral cortical processing is not yet fully understood. In addition, even in the sensory thalamic nuclei such as the posterior nucleus, the core-type cortical afferents of single TCNs show a wide distribution far exceeding the columnar size even in area S1, although the distribution is narrower than that of motor thalamic neurons (Ohno et al., 2012). These findings suggest that the dual, columnar and laminar, organization of TC afferents is limited to the primary sensory thalamic nuclei, but that the majority of TCNs use the laminar organization alone.

The summarized scheme in **Figure 10A** allows the following hypothesis on the local cortical circuitry to be proposed. The blue circuit is likely to serve as a feedforward information-processing system (**Figure 10B**, left). This is strongly supported by well known facts that L4 of area V1 almost exclusively contains simple cells, and contrastingly that L2/3 and L5 mainly comprise complex cells representing information of higher order (Hubel and Wiesel, 1962; Gilbert, 1977). The processed information is further sent to subcortical and/or other cortical regions *via* L2/3 and L5 neurons. Recently, L5 of the rodent motor/frontal cortex has been reported to contain at least two kinds of pyramidal neuron groups: subcerebral projection neurons, including CSNs and corticopontine neurons, and crossed corticostriatal neurons. The two neuron groups differ not only in major projection targets but also in local connections (Morishima et al., 2011; Kiritani et al., 2012). Moreover, L2/3 pyramidal neurons in motor-associated areas should be classified into two types as illustrated in **Figure 6A** (Kaneko et al., 1994b; Cho et al., 2004b); pyramidal neurons forming two axon collateral bushes in L2/3 and L5 are more frequently encountered in lower L2/3 than in upper L2/3, whereas those making only one bush in L2/3 are more numerous in upper L2/3 than in lower L2/3. These findings suggest that the information processing is more complicated than that illustrated with the blue circuit. However, the findings are not contradictory to the concept that the blue circuit is a feedforward information-processing system.

In contrast to the blue circuit in **Figure 10**, the red circuit sends the input information back to the input source site, i.e., the thalamic nuclei, with relative independence from the blue one (**Figure 10B**, right). The corticothalamic projection has generally been considered to work as a feedback circuit, because the thalamic nuclei are the sole input gate for corticopetal information flows, including sensory and motor/cerebellar flows (for review, cf. Alitto and Usrey, 2003), and the best site for feedback control. If CTNs work as a true “feedback” circuit like the circuit of a feedback control system in engineering, the output information of the system, i.e., the information presented by L2/3 and/or L5 pyramidal neurons, should be conveyed back to the input gate *via* CTNs. CTNs, however, receive only weak inputs from L2/3 or L5 neurons (**Figure 10A**). This finding is partly supported in area V1 by the *in vivo* electrophysiological observation that many L6 neurons show simple cell responses (Hubel and Wiesel, 1962; Gilbert, 1977; Martinez et al., 2005), indicating that L6 neurons do not effectively use the information expressed by L2/3 or L5 complex cells. It hence appears necessary to consider other functions for CTNs than the feedback control.

It has long been hypothesized that L6 CTNs, together with TCNs, constitute a recurrent circuit, because of well known phenomena suggesting TC reverberating activity, such as augmenting responses and repetitive discharges in sensory areas (Morison and Dempsey, 1943; Chang, 1950). Augmenting responses are also observed between the VA–VL and area M1 in the rat brain (Castro-Alamancos and Connors, 1996a,b). In addition, TCNs have been proposed to, with the help of thalamic reticular nucleus (Rt) neurons, serve as an oscillation generator in the corticothalamic loop (Buzsáki, 1991; Steriade et al., 1993). On the other hand, although the effect of CTN excitation on TCNs has long been elusive for lack of a CTN-selective stimulation method, recent progress in optogenetic techniques makes it feasible to stimulate CTN axons specifically, and the selective stimulation of CTNs has been shown to evoke clear EPSCs in TCNs and Rt neurons monosynaptically (Cruikshank et al., 2010). Since “modulator” afferents to thalamic relay neurons, the major population of which is L6 CTNs, are known to be far more numerous than subcortical and cortical “driver” afferents to relay neurons (for review, see Sherman and Guillery, 2006), the effect of L6 CTNs on TCNs is considered to be large as an assembly even if unitary EPSCs evoked by single CTN activation are small. Taken together, it is likely that the red circuit in **Figure 10B**, together with the black TC projection, constitutes a dynamical system, where the present state ***x***(*t*) of TCNs has a large effect on the next state ***x***(*t* + *dt*) through CTNs and Rt neurons, and thereby works as a mechanism producing autonomous, self-sustaining activity of the corticothalamic loop. Thus, it is plausible that the two blue and red circuits in **Figure 10** are embedded in the local circuit of the cerebral cortex as the parts of feedforward information-processing and autonomous dynamical systems, respectively.

## Conflict of Interest Statement

The author declares that the research was conducted in the absence of any commercial or financial relationships that could be construed as a potential conflict of interest.

## References

[B1] Akers R. M. Killackey H. P. (1978). Organization of corticocortical connections in the parietal cortex of the rat. *J. Comp. Neurol.* 181 513–537 690276 10.1002/cne.901810305

[B2] Alitto H. J. Usrey W. M. (2003). Corticothalamic feedback and sensory processing. *Curr. Opin. Neurobiol.* 13 440–445 12965291 10.1016/s0959-4388(03)00096-5

[B3] Anderson C. T. Sheets P. L. Kiritani T. Shepherd G. M. (2010). Sublayer-specific microcircuits of corticospinal and corticostriatal neurons in motor cortex. *Nat. Neurosci.* 13 739–744 20436481 10.1038/nn.2538 PMC2876193

[B4] Ayling O. G. Harrison T. C. Boyd J. D. Goroshkov A. Murphy T. H. (2009). Automated light-based mapping of motor cortex by photoactivation of channelrhodopsin-2 transgenic mice. *Nat. Methods* 6 219–224 19219033 10.1038/nmeth.1303

[B5] Bannister A. P. Thomson A. M. (2007). Dynamic properties of excitatory synaptic connections involving layer 4 pyramidal cells in adult rat and cat neocortex. *Cereb. Cortex* 17 2190–2203 17116652 10.1093/cercor/bhl126

[B6] Buhl E. H. Tamás G. Szilágyi T. Stricker C. Paulsen O. Somogyi P. (1997). Effect, number and location of synapses made by single pyramidal cells onto aspiny interneurones of cat visual cortex. *J. Physiol.* 500 689–713 9161986 10.1113/jphysiol.1997.sp022053 PMC1159419

[B7] Buzsáki G. (1991). The thalamic clock: emergent network properties. *Neuroscience* 41 351–364 1870695 10.1016/0306-4522(91)90332-i

[B8] Castro-Alamancos M. A. Connors B. W. (1996a). Spatiotemporal properties of short-term plasticity sensorimotor thalamocortical pathways of the rat. *J. Neurosci.* 16 2767–2779 8786452 10.1523/JNEUROSCI.16-08-02767.1996 PMC6578742

[B9] Castro-Alamancos M. A. Connors B. W. (1996b). Cellular mechanisms of the augmenting response: short-term plasticity in a thalamocortical pathway. *J. Neurosci.* 16 7742–7756 8922430 10.1523/JNEUROSCI.16-23-07742.1996 PMC6579081

[B10] Chagnac-Amitai Y. Connors B. W. (1989). Synchronized excitation and inhibition driven by intrinsically bursting neurons in neocortex. *J. Neurophysiol.* 62 1149–6112 2585046 10.1152/jn.1989.62.5.1149

[B11] Chang H.-T. (1950). The repetitive discharges of corticothalamic reverberating circuit. *J. Neurophysiol.* 13 235–257 15415772 10.1152/jn.1950.13.3.235

[B12] Cho R.-H. Segawa S. Mizuno A. Kaneko T. (2004a). Intracellularly labeled pyramidal neurons in the cortical areas projecting to the spinal cord. I. Electrophysiological properties of pyramidal neurons. *Neurosci. Res.* 50 381–394 15567476 10.1016/j.neures.2004.08.006

[B13] Cho R.-H. Segawa S. Okamoto K. Mizuno A. Kaneko T. (2004b). Intracellularly labeled pyramidal neurons in the cortical areas projecting to the spinal cord. II. Intra- and juxta-columnar projection of pyramidal neurons to corticospinal neurons. *Neurosci. Res.* 50 395–410 15567477 10.1016/j.neures.2004.08.007

[B14] Colebatch J. G. (2007). Bereitschaftspotential and movement-related potentials: origin, significance, and application in disorders of human movement. *Mov. Disord.* 22 601–610 17260337 10.1002/mds.21323

[B15] Connors B. W. Gutnick M. J. Prince D. A. (1982). Electrophysiological properties of neocortical neurons in vitro. *J. Neurophysiol.* 48 1302–1320 6296328 10.1152/jn.1982.48.6.1302

[B16] Cruikshank S. J. Urabe H. Nurmikko A. V. Connors B. W. (2010). Pathway-specific feedforward circuits between thalamus and neocortex revealed by selective optical stimulation of axons. *Neuron* 65 230–245 20152129 10.1016/j.neuron.2009.12.025 PMC2826223

[B17] Dalva M. B. Katz L. C. (1994). Rearrangements of synaptic connections in visual cortex revealed by laser photostimulation. *Science* 265 255–258 7912852 10.1126/science.7912852

[B18] Deuchars J. West D. C. Thomson A. M. (1994). Relationships between morphology and physiology of pyramid–pyramid single axon connections in rat neocortex in vitro. *J. Physiol.* 478 423–435 7965856 10.1113/jphysiol.1994.sp020262 PMC1155663

[B19] Donoghue J. P. Kerman K. L. Ebner F. F. (1979). Evidence for two organizational plans within the somatic sensory-motor cortex of the rat. *J. Comp. Neurol.* 183 647–664 103941 10.1002/cne.901830312

[B20] Donoghue J. P. Wise S. P. (1982). The motor cortex of the rat: cytoarchitecture and microstimulation mapping. *J. Comp. Neurol.* 212 76–88 6294151 10.1002/cne.902120106

[B21] Fujiyama F. Furuta T. Kaneko T. (2001). Immunocytochemical localization of candidates for vesicular glutamate transporters in the rat cerebral cortex. *J. Comp. Neurol.* 435 379–387 11406819 10.1002/cne.1037

[B22] Furuta T. Deschênes M. Kaneko T. (2011). Anisotropic distribution of thalamocortical boutons in barrels. *J. Neurosci.* 31 6432–6439 21525284 10.1523/JNEUROSCI.6154-10.2011 PMC6622683

[B23] Galarreta M. Hestrin S. (1999). A network of fast-spiking cells in the neocortex connected by electrical synapses. *Nature* 402 72–75 10573418 10.1038/47029

[B24] Gilbert C. D. (1977). Laminar differences in receptive field properties of cells in cat primary visual cortex. *J. Physiol.* 268 391–421 874916 10.1113/jphysiol.1977.sp011863 PMC1283670

[B25] Hall R. D. Lindholm E. P. (1974). Organization of motor and somatosensory neocortex in the albino rat. *Brain. Res.* 6 23–38

[B26] Hoffer Z. S. Hoover J. E. Alloway K. D. (2003). Sensorimotor corticocortical projections from rat barrel cortex have an anisotropic organization that facilitates integration of inputs from whiskers in the same row. *J. Comp. Neurol.* 466 525–544 14566947 10.1002/cne.10895

[B27] Hooks B. M. Hires S. A. Zhang Y. X. Huber D. Petreanu L. Svoboda K. (2011). Laminar analysis of excitatory local circuits in vibrissal motor and sensory cortical areas. *PLoS Biol.* 9:e1000572 10.1371/journal.pbio.1000572 PMC3014926 21245906

[B28] Hubel D. H. Wiesel T. N. (1962). Receptive fields, binocular interaction and functional architecture in the cat’s visual cortex. *J. Physiol.* 160 106–154 14449617 10.1113/jphysiol.1962.sp006837 PMC1359523

[B29] Hwa G. G. Avoli M. (1992). Excitatory postsynaptic potentials recorded from regular-spiking cells in layers II/III of rat sensorimotor cortex. *J. Neurophysiol.* 67 728–737 1349636 10.1152/jn.1992.67.3.728

[B30] Jones E. G. (1984). “Laminar distribution of cortical efferent cells,” in *Cerebral Cortex* Vol. 1 *Cellular Components of the Cerebral Cortex* eds. Peters A. Jones E. G. (New York, NY: Plenum Press) 521–553

[B31] Jones E. G. (1998). Viewpoint: the core and matrix of thalamic organization. *Neuroscience* 85 331–345 9622234 10.1016/s0306-4522(97)00581-2

[B32] Jones E. G. (2001). The thalamic matrix and thalamocortical synchrony. *Trends Neurosci.* 24 595–601 11576674 10.1016/s0166-2236(00)01922-6

[B33] Kameda H. Furuta T. Matsuda W. Ohira K. Nakamura K. Hioki H. (2008). Targeting green fluorescent protein to dendritic membrane in central neurons. *Neurosci. Res.* 61 79–91 18342383 10.1016/j.neures.2008.01.014

[B34] Kameda H. Hioki H. Tanaka Y. H. Tanaka T. Sohn J. Sonomura T. (2012). Parvalbumin-producing cortical interneurons receive inhibitory inputs on proximal portions and cortical excitatory inputs on distal dendrites. *Eur. J. Neurosci.* 35 834–854 10.1111/j.1460-9568.2012.08027.x 22429243

[B35] Kaneko T. Caria M. A. Asanuma H. (1994a). Information processing within the motor cortex. I. Responses of morphologically identified motor cortical cells to stimulation of the somatosensory cortex. *J. Comp. Neurol.* 345 161–171 7929897 10.1002/cne.903450202

[B36] Kaneko T. Caria M. A. Asanuma H. (1994b). Information processing within the motor cortex. II. Intracortical connections between neurons receiving somatosensory cortical input and motor output neurons of the cortex. *J. Comp. Neurol.* 345 172–184 7929898 10.1002/cne.903450203

[B37] Kaneko T. Cho R. Li Y. Nomura S. Mizuno N. (2000). Predominant information transfer from layer III pyramidal neurons to corticospinal neurons. *J. Comp. Neurol.* 423 52–65 10861536 10.1002/1096-9861(20000717)423:1<52::aid-cne5>3.0.co;2-f

[B38] Kaneko T. Saeki K. Lee T. Mizuno N. (1996). Improved retrograde axonal transport and subsequent visualization of tetramethylrhodamine (TMR)-dextran amine by means of an acidic injection vehicle and antibodies against TMR. *J. Neurosci. Methods.* 65 157–165 8740593 10.1016/0165-0270(95)00162-x

[B39] Katz L. C. Dalva M. B. (1994). Scanning laser photostimulation: a new approach for analyzing brain circuits. *J. Neurosci. Methods* 54 205–218 7869753 10.1016/0165-0270(94)90194-5

[B40] Killackey H. P. Koralek K. A. Chiaia N. L. Rhodes R. W. (1989). Laminar and areal differences in the origin of the subcortical projection neurons of the rat somatosensory cortex. *J. Comp. Neurol.* 282 428–445 2715391 10.1002/cne.902820309

[B41] Kiritani T. Wickersham I. R. Seung H. S. Shepherd G. M. (2012). Hierarchical connectivity and connection-specific dynamics in the corticospinal–corticostriatal microcircuit in mouse motor cortex. *J. Neurosci.* 32 4992–5001 22492054 10.1523/JNEUROSCI.4759-11.2012 PMC3329752

[B42] Kornhuber H. H. Deecke L. (1965). Hirnpotentialänderungen bei Willkürbewegungen und passiven Bewegungen des Menschen: Bereitschaftspotential und reafferente Potentiale. *Pflügers Arch.* 284 1–17 14341490

[B43] Krieg W. J. (1946). Connections of the cerebral cortex. I. The albino rat. B. Structure of the cortical areas. *J. Comp. Neurol.* 84 277–323 20991808 10.1002/cne.900840302

[B44] Kuramoto E. Fujiyama F. Nakamura K. C. Tanaka Y. Hioki H. Kaneko T. (2011). Complementary distribution of glutamatergic cerebellar and GABAergic basal ganglia afferents to the rat motor thalamic nuclei. *Eur. J. Neurosci.* 33 95–109 21073550 10.1111/j.1460-9568.2010.07481.x

[B45] Kuramoto E. Furuta T. Nakamura K. C. Unzai T. Hioki H. Kaneko T. (2009). Two types of thalamocortical projections from the motor thalamic nuclei of the rat: a single neuron-tracing study using viral vectors. *Cereb. Cortex* 19 2065–2077 19174446 10.1093/cercor/bhn231

[B46] Larkum M. E. Senn W. Luscher H. R. (2004). Top-down dendritic input increases the gain of layer 5 pyramidal neurons. *Cereb. Cortex.* 14 1059–1070 15115747 10.1093/cercor/bhh065

[B47] Lefort S. Tomm C. Floyd Sarria J. C. Petersen C. C. (2009). The excitatory neuronal network of the C2 barrel column in mouse primary somatosensory cortex. *Neuron* 61 301–316 19186171 10.1016/j.neuron.2008.12.020

[B48] Leong S. K. (1983). Localizing the corticospinal neurons in neonatal, developing and mature albino rat. *Brain Res.* 265 1–9 6850310 10.1016/0006-8993(83)91327-6

[B49] Llano D. A. Sherman S. M. (2009). Differences in intrinsic properties and local network connectivity of identified layer 5 and layer 6 adult mouse auditory corticothalamic neurons support a dual corticothalamic projection hypothesis. *Cereb. Cortex* 19 2810–2826 19351905 10.1093/cercor/bhp050 PMC2774389

[B50] Markram H. Tsodyks M. (1996). Redistribution of synaptic efficacy between neocortical pyramidal neurons. *Nature* 382 807–810 8752273 10.1038/382807a0

[B51] Martinez L. M. Wang Q. Reid R. C. Pillai C. Alonso J.-M. Sommer R. T. Hirsch J. A. (2005). Receptive field structure varies with layer in the primary visual cortex. *Nat. Neurosci.* 8 372–379 15711543 10.1038/nn1404 PMC1987328

[B52] Mercer A. West D. C. Morris O. T. Kirchhecker S. Kerkhoff J. E. Thomson A. M. (2005). Excitatory connections made by presynaptic cortico-cortical pyramidal cells in layer 6 of the neocortex. *Cereb. Cortex* 15 1485–1496 15647524 10.1093/cercor/bhi027

[B53] Miller M. W. (1987). The origin of corticospinal projection neurons in rat. *Exp. Brain. Res.* 67 339–351 3622693 10.1007/BF00248554

[B54] Morishima M. Morita K. Kubota Y. Kawaguchi Y. (2011). Highly differentiated projection-specific cortical subnetworks. *J. Neurosci.* 31 10380–10391 21753015 10.1523/JNEUROSCI.0772-11.2011 PMC6623049

[B55] Morison R. S. Dempsey E. W. (1943). Mechanism of thalamocortical augmentation and repetition. *Am. J. Physiol.* 138 297–308

[B56] Neafsey E. J. Bold E. L. Haas G. Hurley-Gius K. M. Quirk G. Sievert C. F. (1986). The organization of the rat motor cortex: a microstimulation mapping study. *Brain Res. Rev.* 11 77–96 10.1016/s0006-8993(86)80191-3 3708387

[B57] Ohana O. Sakmann B. (1998). Transmitter release modulation in nerve terminals of rat neocortical pyramidal cells by intracellular calcium buffers. *J. Physiol.* 513 135–148 9782165 10.1111/j.1469-7793.1998.135by.x PMC2231265

[B58] Ohno S. Kuramoto E. Furuta T. Hioki H. Tanaka Y. R. Fujiyama F. (2012). Morphological analysis of thalamocortical axon fibers of rat posterior thalamic nuclei: a single neuron tracing study with viral vectors. *Cereb. Cortex* 22 2840–2857 22190433 10.1093/cercor/bhr356

[B59] Paxinos G. Watson C. (2007). *The Rat Brain in Stereotaxic Coordinates* 6th Edn. London: Academic Press

[B60] Petreanu L. Mao T. Sternson S. M. Svoboda K. (2009). The subcellular organization of neocortical excitatory connections. *Nature* 457 1142–1145 19151697 10.1038/nature07709 PMC2745650

[B61] Roland P. E. (2002). Dynamic depolarization fields in the cerebral cortex. *Trends Neurosci.* 25 183–190 11998686 10.1016/s0166-2236(00)02125-1

[B62] Sanderson K. J. Welker W. Shambes G. M. (1984). Reevaluation of motor cortex and of sensorimotor overlap in cerebral cortex of albino rats. *Brain. Res.* 292 251–260 6692158 10.1016/0006-8993(84)90761-3

[B63] Shepherd G. M. Stepanyants A. Bureau I. Chklovskii D. Svoboda K. (2005). Geometric and functional organization of cortical circuits. *Nat. Neurosci.* 8 782–790 15880111 10.1038/nn1447

[B64] Shepherd G. M. Svoboda K. (2005). Laminar and columnar organization of ascending excitatory projections to layer 2/3 pyramidal neurons in rat barrel cortex. *J. Neurosci.* 25 5670–5679 15958733 10.1523/JNEUROSCI.1173-05.2005 PMC6724876

[B65] Sherman S. M. Guillery R. G. (2006). *Exploring the Thalamus and its Role in Cortical Function*. Cambridge, MA: The MIT Press

[B66] Silva L. R. Gutnick M. J. Connors B. W. (1991). Laminar distribution of neuronal membrane properties in neocortex of normal and reeler mouse. *J. Neurophysiol.* 66 2034–2040 1812234 10.1152/jn.1991.66.6.2034

[B67] Skoglund T. S. Pascher R. Berthold C. H. (1997). The existence of a layer IV in the rat motor cortex. *Cereb. Cortex* 7 178–180 9087825 10.1093/cercor/7.2.178

[B68] Somogyi P. (1978). The study of Golgi stained cells and of experimental degeneration under the electron microscope: a direct method for the identification in the visual cortex of three successive links in a neuron chain. *Neuroscience* 3 167–180 83552 10.1016/0306-4522(78)90099-4

[B69] Steriade M. McCormick D. A. Sejnowski T. J. (1993). Thalamocortical oscillations in the sleeping and aroused brain. *Science* 262 679–685 8235588 10.1126/science.8235588

[B70] Sutor B. Hablitz J. J. (1989). EPSPs in rat neocortical neurons in vitro. I. Electrophysiological evidence for two distinct EPSPs. *J. Neurophysiol.* 61 607–620 2709103 10.1152/jn.1989.61.3.607

[B71] Tanaka Y. H. Tanaka Y. R. Fujiyama F. Furuta T. Yanagawa Y. Kaneko T. (2011a). Local connections of layer 5 GABAergic interneurons to corticospinal neurons. *Front. Neural Circuits* 5:12 10.3389/fncir.2011.00012 PMC3182329 21994491

[B72] Tanaka Y. R. Tanaka Y. H. Konno M. Fujiyama F. Sonomura T. Okamoto-Furuta K. (2011b). Local connections of excitatory neurons to corticothalamic neurons in the rat barrel cortex. *J. Neurosci.* 31 18223–28236 22171028 10.1523/JNEUROSCI.3139-11.2011 PMC6623880

[B73] Tennant K. A. Adkins D. L. Donlan N. A. Asay A. L. Thomas N. Kleim J. A. (2011). The organization of the forelimb representation of the C57BL/6 mouse motor cortex as defined by intracortical microstimulation and cytoarchitecture. *Cereb. Cortex* 21 865–876 20739477 10.1093/cercor/bhq159 PMC3059888

[B74] Thomson A. M. (1997). Activity-dependent properties of synaptic transmission at two classes of connections made by rat neocortical pyramidal axons in vitro. *J. Physiol.* 502 131–147 9234202 10.1111/j.1469-7793.1997.131bl.x PMC1159577

[B75] Thomson A. M. Bannister A. P. (1998). Postsynaptic pyramidal target selection by descending layer III pyramidal axons: dual intracellular recordings and biocytin filling in slices of rat neocortex. *Neuroscience* 84 669–683 9579775 10.1016/s0306-4522(97)00557-5

[B76] Thomson A. M. Girdlestone D. West D. C. (1988). Voltage-dependent currents prolong single-axon postsynaptic potentials in layer III pyramidal neurons in rat neocortical slices. *J. Neurophysiol.* 60 1896–1907 2906995 10.1152/jn.1988.60.6.1896

[B77] Thomson A. M. West D. C. (1993). Fluctuations in pyramid–pyramid excitatory postsynaptic potentials modified by presynaptic firing pattern and postsynaptic membrane potential using paired intracellular recordings in rat neocortex. *Neuroscience* 54 329–346 8336828 10.1016/0306-4522(93)90256-f

[B78] Thomson A. M. West D. C. Wang Y. Bannister A. P. (2002). Synaptic connections and small circuits involving excitatory and inhibitory neurons in layers 2–5 of adult rat and cat neocortex: triple intracellular recordings and biocytin labelling in vitro. *Cereb. Cortex* 12 936–953 12183393 10.1093/cercor/12.9.936

[B79] Weiler N. Wood L. Yu J. Solla S. A. Shepherd G. M. (2008). Top-down laminar organization of the excitatory network in motor cortex. *Nat. Neurosci.* 11 360–366 18246064 10.1038/nn2049 PMC2748826

[B80] Welker C. (1971). Microelectrode delineation of fine grain somatotopic organization of SmI cerebral neocortex in albino rat. *Brain Res.* 26 259–275 4100672

[B81] Welker E. Hoogland P. V Van der Loos H. (1988). Organization of feedback and feedforward projections of the barrel cortex: a PHA-L study in the mouse. *Exp. Brain Res.* 73 411–435 3215316 10.1007/BF00248234

[B82] West D. C. Mercer A. Kirchhecker S. Morris O. T. Thomson A. M. (2006). Layer 6 cortico-thalamic pyramidal cells preferentially innervate interneurons and generate facilitating EPSPs. *Cereb. Cortex* 16 200–211 15843627 10.1093/cercor/bhi098

[B83] White E. L. (1989). *Cortical Circuits: Synaptic Organization of the Cerebral Cortex – Structure, Function and Theory*. Boston, MA: Birkhäuser

[B84] White E. L. Hersch S. M. (1981). Thalamocortical synapses of pyramidal cells which project from SmI to MsI cortex in the mouse. *J. Comp. Neurol.* 198 167–181 7229139 10.1002/cne.901980114

[B85] White E. L. Keller A. (1987). Intrinsic circuitry involving the local axon collaterals of corticothalamic projection cells in mouse SmI cortex. *J. Comp. Neurol.* 262 13–26 3624546 10.1002/cne.902620103

[B86] Wise S. P. Jones E. G. (1977). Cells of origin and terminal distribution of descending projections of the rat somatic sensory cortex. *J. Comp. Neurol.* 175 129–157 408380 10.1002/cne.901750202

[B87] Wood L. Gray N. W. Zhou Z. Greenberg M. E. Shepherd G. M. (2009). Synaptic circuit abnormalities of motor–frontal layer 2/3 pyramidal neurons in an RNA interference model of methyl-CpG-binding protein 2 deficiency. *J. Neurosci.* 29 12440–12448 19812320 10.1523/JNEUROSCI.3321-09.2009 PMC2782478

[B88] Wood L. Shepherd G. M. (2010). Synaptic circuit abnormalities of motor–frontal layer 2/3 pyramidal neurons in a mutant mouse model of Rett syndrome. *Neurobiol. Dis.* 38 281–287 20138994 10.1016/j.nbd.2010.01.018 PMC2854239

[B89] Yu J. Anderson C. T. Kiritani T. Sheets P. L. Wokosin D. L. Wood L. (2008). Local-circuit phenotypes of layer 5 neurons in motor–frontal cortex of YFP-H mice. *Front. Neural Circuits* 2:6 10.3389/neuro.04.006.2008 PMC2614859 19129938

[B90] Zarrinpar A. Callaway E. M. (2006). Local connections to specific types of layer 6 neurons in the rat visual cortex. *J. Neurophysiol.* 95 1751–1761 16319201 10.1152/jn.00974.2005

[B91] Zhang Z. W Deschênes M. (1997). Intracortical axonal projections of lamina VI cells of the primary somatosensory cortex in the rat: a single-cell labeling study. *J. Neurosci.* 17 6365–6379 9236245 10.1523/JNEUROSCI.17-16-06365.1997 PMC6568349

